# The Influence of Vitamin A Supplementation on Iron Status

**DOI:** 10.3390/nu5114399

**Published:** 2013-11-07

**Authors:** Fernanda B. Michelazzo, Julicristie M. Oliveira, Juliana Stefanello, Liania A. Luzia, Patricia H. C. Rondó

**Affiliations:** 1 Department of Food Science and Experimental Nutrition, School of Pharmacy, University of São Paulo, São Paulo 05508-900, Brazil; E-Mail: nutrimichelazzo@hotmail.com; 2 School of Applied Sciences, University of Campinas, Limeira 13484-350, Brazil; E-Mail: julicristie.oliveira@fca.unicamp.br; 3 Department of Maternal-Infant Nursing and Public Health, College of Nursing, University of São Paulo, Ribeirão Preto 14040-902, Brazil; E-Mail: julistello@yahoo.com.br; 4 Department of Nutrition, School of Public Health, University of São Paulo, São Paulo 01246-904, Brazil; E-Mail: lianialuzia@usp.br

**Keywords:** vitamin A, iron, anemia, iron deficiency anemia, micronutrients

## Abstract

Vitamin A (VA) and iron deficiencies are important nutritional problems, affecting particularly preschool children, as well as pregnant and lactating women. A PubMed (National Library of Medicine, National Institutes of Health, Bethesda, MD, USA) literature review was carried out to search for clinical trials published from 1992 to 2013 that assessed the influence of vitamin A supplementation on iron status. Simultaneous use of iron and vitamin A supplements seemed to be more effective to prevent iron deficiency anemia than the use of these micronutrients alone. Some studies did not include a placebo group and only a few of them assessed vitamin A status of the individuals at baseline. Moreover, the studies did not consider any inflammatory marker and a reasonable number of iron parameters. Another important limitation was the lack of assessment of hemoglobin variants, especially in regions with a high prevalence of anemia. Assessment of hemoglobin variants, inflammatory markers and anemia of chronic inflammation would be important to the studies investigated. Studies involving different populations are necessary to elucidate the interaction between the two micronutrients, especially regarding iron absorption and modulation of erythropoiesis.

## 1. Introduction

Vitamin A (VA) and iron (Fe) deficiencies are important nutritional problems, affecting particularly preschool children, as well as pregnant and lactating women [1,2,3,4].

As early as 1922, Findlay and Mackenzie [5] investigated the changes caused by the administration of a VA-deficient diet on the hematopoietic tissue of healthy young rats. Areas of gelatinous degeneration were observed in the femur of the animals dying from vitamin A deficiency (VAD), whereas in animals surviving for a longer period of time the hematopoietic tissue was almost completely replaced by fibrosis tissue stroma. Over the subsequent years, several studies have demonstrated the association between VAD and hematopoietic cell alterations followed by iron deficiency anemia, although some reports are contradictory [4,6,7].

Fe differs from other minerals because its balance in the human body is regulated by absorption, in view that there is no physiological mechanism for excretion. VA can affect several stages of Fe metabolism, which include erythropoiesis and the release of Fe from ferritin stores [8]. Fe deficiency anemia, which develops in a series of steps starting with the depletion of Fe stores, is identified by a reduction in serum Fe, an increase in total iron binding capacity (TIBC) and transferrin receptors, low transferrin saturation, reduced serum ferritin, low mean corpuscular volume (MCV) and low mean corpuscular hemoglobin (MCH) [1]. In contrast, VAD anemia is associated with a reduction in serum Fe, low TIBC, low transferrin saturation, and increased serum ferritin concentration, due to a lower mobilization of Fe stores, with increased deposition of Fe in the liver and spleen [1,6].

A review based on clinical trial studies was conducted to address the influence of vitamin A supplementation on iron status and to identify areas that require further research.

## 2. Materials and Methods

A PubMed (National Library of Medicine, National Institutes of Health, Bethesda, MD, USA) search using the following strategy: (“vitamin A”[mesh] AND (“anemia”[MeSH Terms] OR “iron”[MeSH Terms])) OR (“vitamin A”[title] AND (anemia [title] OR iron [title])) AND (clinical trial) AND (“1992/05/31”[PDat]: “2013/08/20”[PDat]) was carried out from September 1992 to August 2013. One hundred and eight (108) studies published in the last 21 years were selected from the search. The exclusion criteria were: vitamin A supplementation together with several micronutrients; individuals with acute or chronic diseases; and studies that did not consider the effect of vitamin A supplementation or fortification on iron status. Therefore, based on the main purpose of the review, 14 studies were included. For more information of distribution of the 14 reviewed studies according to country of origin, please see the supplementary files.

### 2.1. Clinical Trials

Clinical trials have been conducted in order to evaluate the effect of VA supplementation/fortification on hematological indicators of Fe parameters. Some studies have also compared the effect of combined supplementation/fortification with VA and Fe to that of supplementation/fortification with vitamin A or Fe alone. Several aspects of the studies have been discussed: randomization; inclusion of a placebo group; VA assessment at baseline; measurement of an inflammatory marker; and number of Fe parameters evaluated. The main results of the studies are summarize in Table 1 (significant results) and Table 2 (no significant results), following the order as they have been discussed in the text.

#### 2.1.1. Vitamin A Supplementation in Children and Adolescents

Mwanri *et al*. [6] conducted a randomized placebo-controlled clinical trial with 135 anemic (hemoglobin-Hb < 120 g/L) schoolchildren (9–12 year) in Tanzania who received supplements of 5000 IU VA (1.5 mg retinyl acetate), VA (5000 IU) + Fe (200 mg ferrous sulfate), Fe (200 mg), or placebo, 3 days a week for 3 months. Supplementation significantly increased Hb concentrations, with the largest increase being observed in the group supplemented with Fe + VA, in which 88% of the children were no longer anemic compared to only 3% improvement in the placebo group. Although the children did not present at baseline any clinical sign of VAD, their VA status was not investigated, limiting the interpretation of the findings. In addition, the prevalence of anemia was assessed only by Hb, a universally accepted parameter to predict anemia, but with a low specificity and sensitivity to assess the nutritional status of Fe. Additionally, Hb concentrations in children can be modified with age, especially among teenagers exhibiting significant differences in the pattern of changes between gender [9], considering that some girls may have gone through menarche. Other limitations of the study are lack of investigation of Hb variants and an inflammatory marker.

In a randomized, double-blind, controlled trial study carried out by Varma *et al.* [10], 516 Indian children aged 3–5.5 year participating in the Integrated Child Development Service (ICDS) received for 6 months either a non-fortified or a fortified “premix” with VA (500 IU as retinyl acetate), Fe (14 mg as ferrous fumarate), and folic acid (50 μg) added to prepare *khichdi* (rice and lentils mixture), to decrease the prevalence of Fe and VA deficiencies. There were significant differences in the prevalence of anemia, iron deficiency anemia, mean serum retinol concentration, and C-reactive protein (CRP) in both groups of children at baseline. The prevalence of VAD (serum retinol < 0.7 μmol/L) was 35% in the non-fortified group and 43% in the fortified group. After a subgroup analysis with anemic children at baseline, Hb concentrations increased significantly in the fortified group compared with the non-fortified group, from weeks 0 to 24 (from 99.9 to 116.9 g/L *vs.* 98.9 to 109.9 g/L, respectively; *p* < 0.04). After 24 weeks, serum ferritin was significantly higher in the fortified group (from 25.1 to 35.5 μg/L) than in the non-fortified group (from 25.7 to 22.9 μg/L; *p* < 0.001). In this study [10], the concentration of CRP was determined at baseline, although it was not utilized as a confounder in the statistical analysis. The use of only one intervention group limited the interpretation of the results, because it was not possible to determine the isolated impact of each micronutrient on anemia and iron deficiency anemia.

**Table 1:** Clinical trials that showed a significant impact of vitamin A supplementation/fortification alone or in combination with iron, folic acid, vitamin C and riboflavin on iron status.

References	Country	Population (Age in Years)	*N*	Intervention (Groups)	Time (Month)	Impact	Conclusions
*Children and Adolescents*							
Mwanri *et al.* (2000) [6]	Tanzania	Anemic children (9–12)	135	5000 IU VA/3× week; 5000 IU VA + 200 mg Fe/3× week; 200 mg Fe/3× week; Placebo	3	↑Hb = 13.5; ↑Hb = 22.1; ↑Hb = 17.5; ↑Hb = 3.6	↑Hb in the Fe + VA group (p < 0.05)
Varma *et al.* (2007) [10]	India	Children (3–5.5)	516	Rice and lentils fortified with 500 IU VA + 14 mg Fe + 50 μg folic acid; 6 times/week; Placebo	6	↑Hb = 4.0, ↑serum ferritin = 10.4; ↑Hb = 4.0, ↓serum ferritin = −2.8	↑serum ferritin in the VA + Fe + folic acid group (p < 0.001)
Zimmermann *et al.* (2006) [11]	Morocco	Schoolchildren (5–13)	81	200,000 IU VA ^†^ at baseline and after 5 months; Placebo	10	↑Hb = 7.0, MCV = 7.0, serum ferritin = −7.0, ↓TfR = −2.3, EPO = 6.9, ZnPP = -4.0; ↑Hb = 1.0, MCV = 0.0, serum ferritin = 1.0, ↓TfR = −0.2, EPO = 3.3, ZnPP = 1.0	↑Hb, MCV and EPO in the VA group (p < 0.02)
Kapil *et al.* (2005) [3]	India	Adolescent girls (17–18)	39	200,000 IU VA ^†^ + 100 mg Fe + 500 μg folic acid + 60 mg vitamin C/day; 100 mg Fe + 500 μg folic acid + 60 mg vitamin C/day	3.3	↑Hb = 18; ↑Hb = 13	↑Hb status in both groups (p < 0.05); higher in the VA group
*Children and Adolescents*							
Leenstra *et al.* (2009) [12]	Kenya	Anemic adolescent girls (12–18)	249	25,000 IU VA + 120 mg Fe/week; 25,000 IU VA + Placebo/week; 120 mg Fe/week + Placebo; Placebo/week	5	VA-supplemented group compared to vitamin A placebo group (adjusted for Fe supplementation): ↓Hb = −0.7, ↓serum ferritin = −1.7; Fe-supplemented group compared to Fe placebo group (adjusted for vitamin A supplementation): ↑Hb = 5.2, ↑serum ferritin = 13.3	↑Hb and serum ferritin (p < 0.001) only in the Fe supplemented groups
*Pregnant and Lactating Women*							
Suharno *et al.* (1993) [13]	Indonesia	Pregnant women (17–35)	251	8000 IU VA + 60 mg Fe/day; 8000 IU VA + Fe placebo/day; 60 mg Fe/day + vitamin A placebo; Placebo	2	↑Hb = 12.70, Ht = 0.04, ↑ serum ferritin = 1.82, ↑TS = 0.036, ↑serum iron = 1.62, ↓TIBC= −3.00; ↑Hb = 3.68, Ht = 0.01, ↑serum ferritin = 1.34, ↑TS = 0.006, ↑serum iron = 0.22, ↓TIBC = −0.60; ↑Hb = 7.71, Ht = 0.02, ↑serum ferritin = 2.22, ↑TS = 0.017, ↑serum iron = 0.81, ↓TIBC = −1.30; ↑Hb = 2.00, Ht = 0.01, ↑serum ferritin = 1.22, ↑TS = 0.002, ↑serum iron = 0.10, ↓TIBC = −0.10	Difference in all parameters between the VA + Fe group and the other groups (p < 0.001)
*Pregnant and Lactating Women*							
Muslimatun *et al.* (2001a, 2001b) [14,15]	Indonesia	Pregnant women (17–35)	190	20,000 IU VA + 120 mg Fe + 500μg folic acid/week; 120 mg Fe + 500 μg folic acid/week; 90–120 mg Fe + 250 μg folic acid ^††^/day	5	↑Hb = 3.70, ↓serum ferritin = −7.10, ↑TfR = 0.43; ↑Hb = 2.10, ↓serum ferritin = −3.00, ↑TfR = 0.47; ↓Hb = −0.70, ↓ serum ferritin = −5.30, ↑TfR = 0.56	Difference in Hb (p < 0.05), serum ferritin, TfR (p < 0.01) between the VA + Fe + folic acid group and the other groups
Tanumihardjo (2002) [16]	Indonesia	Pregnant women (18–37)	27	8000 IU VA/day; 60 mg Fe/day; 8000 IU VA + 60 mg Fe/day; Placebo	2	↑Hb = 7.10, ↑Ht = 0.036, ↑serum ferritin = 4.70; ↑Hb = 6.60, ↑Ht = 0.018, ↑serum ferritin = 15.00; ↑Hb = 3.90, ↑Ht = 0.049, ↑serum ferritin = 12.00; ↓Hb = −9.00, ↓Ht = −0.034, ↓serum ferritin = −13.80	Positive effect of supplementation with VA + Fe on indicators of iron status (p < 0.05)
Suprapto *et al.* (2002) [17]	Indonesia	Anemic pregnant women (<35)	84	5000 IU VA + 60 mg Fe + 250 μg folic acid + 5 mg riboflavin; 5000 IU VA + 60 mg Fe + 250 μg folic acid; 60 mg Fe + 250 μg folic acid + 5 mg riboflavin; 60 mg Fe + 250 μg folic acid + placebo	2	↑Hb = 4.6; ↑Hb = 1.9; ↑Hb = 8.2; ↑Hb = 4.9	Increase in Hb in all groups (p < 0.05), except in the VA + Fe + folic acid group (p > 0.05)
Sun *et al.* (2010) [18]	China	Anemic pregnant women (20–30)	180	6000 IU VA+ 60 mg Fe+ 400 μg folic acid/day; 60 mg Fe/day; 60 mg Fe+ 400 μg folic acid/day; Placebo	2	↑Hb = 16.5, ↑serum ferritin = 8.12; ↑Hb = 17.9, ↑serum ferritin = 2.11; ↑Hb = 14.7, ↑serum ferritin = 3.38; ↓Hb = −1.98, ↓serum ferritin = −1.61	VA + Fe supplementation was more beneficial to improve iron status and lymphocyte proliferation in pregnancy than Fe alone (p < 0.001)

*N* = sample size; VA = vitamin A retinyl acetate; VA ^†^ = vitamin A retinyl palmitate (international units—IU); Fe = elementary iron; Hb = hemoglobin (g/L); Serum ferritin (μg/L); Retinol = serum retinol (μmol/L); MCV = mean corpuscular volume (fL); RBP = retinol binding protein (mg/L); Prealbumin (mg/L); EPO = erythropoietin (IU/L); TfR = transferrin receptor (mg/L); ZnPP = zinc protoprphyrin (μmol/mol heme); TS = transferrin saturation; Ht = hematocrit (vol/vol); Serum iron (μmol/L); TIBC = total iron-binding capacity (μmol/L); ^††^ = Free access to iron tablets from the Indonesian Governmental Health Service; RDR = relative dose response (mol/mol).

**Table 2:** Clinical trials that showed no significant impact of vitamin A supplementation/fortification alone or in combination with iron, folic acid and vitamin C on iron status.

References	Country	Population (Age in Years)	*N*	Intervention (Groups)	Time (Months)	Impact				Conclusions
*Children and Adolescents*										
Pereira *et al.* (2007) [7]	Brazil	Children and Adolescents (6–14)	267	10,000 IU VA + 40 mg Fe/week; 40 mg Fe/week	7.5	↑Hb = 8.0, ↓anemia = 43.8%, ↑MCV = 1.4, microcytosis = 3.8; ↑Hb = 9.0, ↓anemia = 30.7%, ↑MCV = 1.6, microcytosis = 3.2				No differences between the groups according to mean Hb and prevalence of anemia.
Soekarjo *et al.* (2004) [19]	Indonesia	Adolescents (12–15)	3616	10,000 IU VA/week; 10,000 IU VA + 60 mg Fe/week; 60 mg Fe + 250 μg folic acid/week; Control	3.5	Girls		Boys		No differences among the groups (*p* > 0.05).
						Prepuberal	Puberal	Prepuberal	Puberal	
						↑Hb = 5.9	↑Hb = 2.7	↑Hb = 8.4	↑Hb = 12.0	
						↑Hb = 10.2	↑Hb = 4.4	↑Hb = 7.1	↑Hb = 12.9	
						↑Hb = 7.5	↑Hb = 7.8	↑Hb = 5.3	↑Hb = 7.4	
						↑Hb = 9.0	↑Hb = 5.6	↑Hb = 7.5	↑Hb = 9.8	
Davidsson *et al*. (2003) [20]	Côte d’Ivoire	Schoolchildren (6–13)	13	2.0 mg Fe added to maize porridge; 2.0 mg Fe + 3300 IU VA added to maize porridge	0.7	↓Fe stable isotope in erythrocyte = −1.4				VA added to the meal decreased erythrocyte incorporation of Fe in children in the VA group, but had no impact after a mega dose of VA.
*Pregnant and Lactating Women*										
Semba *et al.* (2001) [21]	Malawi	Pregnant women (20–26)	137	10,000 VA + 30 mg Fe + 400 μg folic acid/day; 30 mg Fe + 400 μg folic acid/day	3.75	↑Hb = 4.7, ↑EPO = 2.39; ↑Hb = 7.3, ↓EPO = −2.87				No difference between the groups.

*N* = sample size; VA=vitamin A (international units—IU); Fe = elementary iron; Hb = hemoglobin (g/L); MCV = mean corpuscular volume (fL); anemia (%); microcytosis (%); Retinol = serum retinol (μmol/L); EPO = erythropoietin (IU/L); Serum ferritin (μg/L); TfR = transferrin receptor (mg/L).

The efficacy of weekly Fe with or without VA supplementation was tested by Pereira *et al.* [7] in a non-placebo-controlled trial. Brazilian schoolchildren (*n* = 267, 6–14 years) were randomly allocated in the following groups: VA (10,000 IU) + Fe (40 mg) or Fe (40 mg) alone. After 7.5 months of supplementation, anemia prevalence was significantly reduced in the Fe group (from 48.4% to 17.7%; *p* < 0.001) and in the VA + Fe group (from 58.1% to 14.3%; *p* < 0.001), but there was no difference between the groups (*p* = 0.48). MCV was also enhanced in the Fe group (from 85.3 to 86.9 fL; *p* < 0.001) and in the Fe + VA group (from 86.3 to 87.7 fL; *p* < 0.001), with no difference between the groups (*p* = 0.64). The prevalence of VAD was not investigated in the baseline of the study; thus, the impact of VA supplementation cannot be accurately interpreted. The authors did not assess inflammatory markers.

Zimmermann *et al.* [11] conducted a double-blind, randomized trial to assess the effects of VA supplementation or placebo, during periods of 5 and 10 months, on 81 Moroccan schoolchildren. No significant differences were observed between the groups at baseline, including serum retinol concentrations. The VA and placebo groups presented, respectively, prevalences of 8% and 6% of VAD and 30% and 32% of low serum retinol (<1.05 μmol/L). The authors observed a significant increase in geometric mean erythropoietin, Hb and MCV (*p* < 0.05) in the VA-supplemented group. There were significant decreases in the prevalence of anemia, transferrin saturation, transferrin receptor-TfR, and in the slope of the regression line of log erythropoietin on Hb geometric mean (*p* < 0.001). There were no differences for zinc protoporphyrin-ZnPP and total Fe body (transferrin receptor: serum ferritin). Besides the influence of VA on erythropoietin, there were also effects of VA on some hormones and cytokines involved in erythropoiesis. VA metabolites regulate transcription of many hepatic genes, and the authors of the study suggest that it could additionally modulate synthesis or catabolism of proteins involved in hepatic Fe stores and mobilization. In this trial, the influence of VA supplementation on the iron parameters investigated (Hb, MCV, serum ferritin, serum TfR, ZnPP, and total body iron) was monitored during follow-up by CRP concentrations. However, the authors did not investigate the prevalence of Hb variants in this population, an important problem in Africa [22].

The three following studies [3,12,19] evaluated the effect of VA supplementation alone or combined with other micronutrients on Hb and other Fe parameters of adolescents.

Kapil *et al.* [3] conducted a randomized double-blind trial to evaluate the effect of supplementation with VA (200,000 IU) + Fe (100 mg) + folic acid (500 μg) + vitamin C (60 mg) or supplementation with Fe (100 mg) + folic acid (500 μg) + vitamin C (60 mg) on Hb concentrations of 39 Indian adolescent girls, aged 17–18 years. Daily supplementation during 100 days (3.3 months) resulted in increased Hb concentrations among adolescent girls in both groups. However, the increase was 5g/L higher in the group that received the mega dose of VA compared with the group without VA. VA status and inflammatory markers were not monitored, and a placebo group was not included in the trial. Moreover, only hemoglobin concentrations were investigated at baseline; therefore, interpretation of the results should be taken with caution.

In a school-based randomized clinical trial conducted by Soekarjo *et al.* [19], 3616 adolescents aged 12 to 15 years from rural and urban areas of Java, Indonesia, received weekly supplementation of VA (10,000 IU), VA (10,000 IU) + Fe (60 mg) or Fe (60 mg) + folic acid (250 μg) for 3.5 months and were compared to a control group. At baseline, the prevalence of VAD varied from 4.4% to 9.3% in girls, and from 6.5% to 14.1% in boys, but the differences were not significant. Anemic pubertal adolescent girls supplemented with Fe + folic acid showed a larger increase in Hb than those receiving only VA (7.8 *vs.* 2.7 g/L, *p* < 0.05). However, no differences in Hb concentrations were observed for anemic boys in the four groups investigated. During the fasting months and holidays, supplements had to be taken at home, and the authors assumed that duration of supplementation was only 9 weeks, which was likely too a short period of time for the supplements to show any effect. Other limitations of the study are lack of investigation of an inflammatory marker and other Fe parameters.

Leenstra *et al.* [12] assessed the effects of weekly VA (25,000 IU) and Fe (120 mg) supplementation in a double-blind, randomized placebo-controlled clinical trial using a factorial design. The study involved 249 (12–18 year) schoolgirls in Kenya with mild/moderate anemia (70–120 g/L) allocated in the following weekly supplementation regimes: VA + Fe; VA + placebo; Fe + placebo or two placebos for 5 months. VA deficiency prevalence was <10% in all groups at baseline. There was no evidence of interactions between VA and Fe supplementation, assessed by Hb, ferritin, and retinol-binding protein (RBP) concentrations. Fe status at baseline significantly modified the effect of Fe supplementation on Hb and serum ferritin (*p* < 0.001), but did not modify the effects of VA. There was insufficient power to assess if the supplementation regimens were modified by VA status at baseline. Blood concentration of CRP was determined at baseline, but there was no subgroup analysis considering the influence of this inflammatory marker in adolescents with and without anemia. The authors did not assess Hb variants.

Davidsson *et al.* [20] evaluated the influence of retinyl palmitate added to Fe-fortified maize porridge on erythrocyte incorporation of Fe in children with vitamin A deficiency, before and after vitamin A supplementation. Erythrocyte incorporation of Fe stable isotopes was measured 14 days after intake of maize porridge (2.0 mg Fe added as ferrous sulfate) with and without added retinyl palmitate (3.5 mmol; 3300 IU). The children were divided in two groups to receive the meals. Retinyl palmitate added to the labeled meal had no positive effect on erythrocyte incorporation of Fe, with Fe incorporation being significantly higher for meals not containing retinyl palmitate. In fact, supplementation with retinyl palmitate decreased erythrocyte incorporation of Fe stable isotopes. At the end of the study, the children were supplemented with a single dose of VA (200,000 IU) and 3 weeks after supplementation the difference in Fe incorporation was no longer present, indicating a possible action of VA on the mobilization and availability of Fe, although the result was not statistically significant. The difference in response to retinyl palmitate added to Fe-fortified maize porridge on erythrocyte incorporation of Fe before and after intake of the vitamin A capsule indicates, indirectly, changes in vitamin A status not measurable by the modified relative dose response (MRDR) technique. The lack of conclusive data on the effect of retinyl palmitate on Fe absorption indicates the complexity of the interactions between vitamin A status, dietary vitamin A and Fe metabolism.

#### 2.1.2. Vitamin A Supplementation in Pregnant and Lactating Women

In a randomized, double-blind, placebo-controlled trial involving pregnant women from Indonesia, Suharno *et al.* [13] selected 251 women that were submitted to four daily supplementation regimens: VA (8000 IU) + Fe (60 mg) (*n* = 63), VA (8000 IU) + placebo (*n* = 63), Fe (60 mg) + placebo (*n* = 63), and placebo (*n* = 62). Mean serum retinol concentrations at baseline varied from 1.04 to 1.11 μmol/L among groups, and 10% of the whole population had VAD. After 2 months of supplementation, the largest increase in Hb was observed for the group supplemented with VA and Fe, reaching a level of 12.78 g/L, followed by the Fe group with 7.71 g/L and the VA group with 3.68 g/L. There were also statistically significant increases in serum ferritin and serum iron, and decreases in TIBC. After the intervention 97% in the VA + Fe group, 68% in the Fe group, and 35% in the VA group women were no longer anemic. Although the authors carefully evaluated the VA status at baseline, no inflammatory marker was considered.

Muslimatun *et al.* [14,15] in a randomized double-blind community-based trial involving 190 pregnant women (16–20 weeks of gestation), 17–35 years of age, compared the effects of weekly supplementation, for 5 months, with VA (20,000 IU) + Fe (120 mg) + folic acid (500 μg), and Fe (120 mg) + folic acid (500 μg), to that of daily Fe (90–120 mg) + folic acid (250 μg). Prevalences of VAD were 13%, 17% and 17% in the weekly VA + Fe + folic acid, Fe + folic acid, and daily Fe + folic acid supplemented groups, respectively. Among the women who were anemic at the beginning of the study, increases in Hb concentration were observed in the weekly VA + Fe + folic acid (10.7 ± 2.3 g/L), weekly Fe + folic acid (6.6 ± 2.3 g/L) and daily Fe + folic acid (3.4 ± 2.6 g/L) groups. Serum ferritin decreased significantly in the group receiving VA + Fe + folic acid compared to the daily Fe + folic acid group, indicating that VA probably improved utilization of iron for hematopoiesis. However, despite a higher serum Fe concentration in the group receiving VA + Fe + folic acid, compared to the other two groups, the difference was not significant. Transferrin receptor concentrations significantly increased in the three groups.

Semba *et al.* [21] evaluated the impact of VA (10,000 IU) + Fe (30 mg) + folic acid (400 μg) or Fe (30 mg) + folic acid (400 μg) supplementation given to 137 pregnant women in Malawi. This was a randomized, double-blind, controlled clinical trial, and at enrolment, 50% of the women were anemic (Hb < 110 g/L). Prevalence of VAD and mean plasma retinol concentration were respectively 34.8% and 0.91 μmol/L in the Fe + folic acid group and 24.7% and 0.81 μmol/L in the VA + Fe + folic acid group (*p* < 0.05). Mean change in Hb concentration increased in the VA + Fe + folic acid (4.7 g/L, *p* = 0.003) and in the Fe + folic acid (7.3 g/L, *p* = 0.003) groups. However, there were no differences between groups in relation to Hb and plasma retinol. No differences were observed in mean concentrations of erythropoietin from baseline to 38 weeks of gestation in the VA + Fe + folic acid (2.39 IU/L, *p* = 0.63) and in the Fe + folic acid (2.87 IU/L, *p* = 0.46) groups. Moreover, no impact was observed in the slope of the regression line between log erythropoietin and Hb. Acute phase proteins (α1-acid glycoprotein and CRP) were analyzed in this trial and considered in the linear regression analysis, but these markers did not influence the effects of VA supplementation on Hb and erythropoietin. Other factors could be associated with the lack of impact of VA supplementation in the pregnant women. Firstly, malaria is endemic in Malawi [23], and a recognized cause of anemia [24]. Secondly, Africa is a continent with high a prevalence of Hb variants [24]. These two epidemiologic and genetic characteristics are important confounders that were not considered in the analysis.

In a study conducted by Tanumihardjo [16], 27 Indonesian women, 18–37 years of age, during the second trimester of gestation were divided into four groups to receive the following supplements: VA (8000 IU); Fe (60 mg); VA (8000 IU) + Fe (60 mg); and placebo. The author observed significant increases in Hb and hematocrit (Ht) in the VA and VA + Fe groups (*p* < 0.05). There was a significant increase in the concentrations of serum ferritin (*p* < 0.002) in the VA + Fe group. In this study an accurate method for assessment of VA status was used: MRDR (modified relative dose response test). However, the author did not investigate inflammatory markers.

Suprapto *et al.* [17] evaluated the impact of adding low dose of VA (5000 IU) together with other micronutrients as a routine supplementation for 84 anemic Indonesian pregnant women (Hb < 110 g/L) in a placebo-controlled clinical trial. The women were allocated into the following groups: VA (5000 IU) + Fe (60 mg) + folic acid (250 μg) + riboflavin (5 mg); VA (5000 IU) + Fe (60 mg) + folic acid (250 μg); Fe (60 mg) + folic acid (250 μg) + riboflavin (5 mg); Fe (60 mg) + folic acid (250 μg) + placebo. The supplements were administered seven days a week for two months, in a double-blind manner. After supplementation, all groups exhibited a significant increase in Hb concentration (*p* < 0.05), except in the VA + Fe + folic acid group. The effect of the different supplementation regimes were analyzed by Hb increment, considering that only Hb was assessed at baseline. The authors did not assess vitamin A concentrations at baseline, and no inflammatory marker.

Prevalence of anemia in Asia is among the highest in the world. In a recent double-blind randomized trial conducted by Sun *et al.* [18] over two months, 180 pregnant Asian women with Hb from 80 to 110 g/L were supplemented daily, according to the following groups: (1) (*n* = 46) VA (6000 IU) + Fe (60 mg) + folic acid (400 μg); (2) (*n* = 47) Fe (60 mg); (3) (*n* = 46) Fe (60 mg) + folic acid (400 μg); and (4) (*n* = 47) placebo. The authors concluded that VA combined with Fe supplementation was more beneficial to improve iron status during pregnancy than iron alone. However, the authors did not consider inflammatory markers assessment and VA measurements at baseline.

## 3. Discussion

Clinical trials involving children, adolescents, pregnant and lactating women were developed in different regions of the world and most of them reported a significant impact of VA supplementation/fortification on Hb and other iron parameters [3,6,10,11,12,13,14,15,16,17,18]. However, the effect of VA supplementation/fortification on iron status should be taken with caution due to differences between populations in terms of VA and Fe status, age of the individuals, type of supplementation or fortification, and duration of the intervention.

All the studies used randomization to allocate the subjects for supplementation or fortification. In spite of the methodological qualities, some of them did not use a placebo group [3,7,14,15] and did not assess the VA status of the subjects at baseline [3,6,7,11,17,18,20]. One study did not assess Fe parameters at baseline [20]. Only few studies included a reasonable number of iron parameters [11,13,14,15]. Another important limitation was the lack of assessment of Hb variants and malaria, especially in regions with a high prevalence of these problems [6,11,12,18,20]. Some studies did not assess the effect of VA supplementation or fortification alone on iron status, but associated to iron and/or folic acid, riboflavin and vitamin C, limiting the interpretation, because it was not possible to determine the isolated impact of each micronutrient on anemia and iron deficiency anemia. This difficulty was more accentuated in studies where hemoglobin was the only parameter assessed [3,6,17].

Thurnham [25] hypothesized that VA supplementation reduces the prevalence of anemia and promotes beneficial effects on morbidity and/or inflammation. This possibility was also considered by Zimmermann *et al.* [11] who proposed three mechanisms to explain the influence of VA status on Fe deficiency anemia: (1) increased resistance to infection and, consequently, to anemia secondary to infection; (2) effects on Fe absorption and/or metabolism; and (3) direct modulation of erythropoiesis.

VA plays an important role in immune function, probably as a result of enhanced antibody production, lymphocyte proliferation, maintenance of the integrity of the mucosal epithelia, and increased T-cell lymphopoiesis [26]. In this review, individuals with acute and/or chronic diseases were excluded. However, it would be important to have determination of inflammatory markers in these studies, considering that subclinical infection may be a problem in developing countries, where iron and VC deficiencies are highly prevalent. Few studies [10,11,12,18] included an inflammatory marker as a confounder in the statistical analysis, or monitored the occurrence of infectious diseases during the follow-up [11,12]. This is an important limitation of these investigations, considering that infectious diseases, blood loss, and diet are the three most important causes of anemia, in view of the synergy between inflammation and bioavailability of dietary iron. Thus, studies assessing the effects of multiple micronutrients on Hb and other iron parameters should considered the possibility of anemia of chronic inflammation [24].

The role of VA in the recovery of nutritional anemia is possibly related to its modulation in the later stages of erythropoiesis [27], erythropoietin synthesis, and transcription of many hepatic genes [11]. Moreover, it is possible that VA status may influence synthesis or catabolism of proteins involved on hepatic Fe storage and mobilization [11,28,29]. Two studies assessed the impact of VA supplementation on Hb, erythropoietin concentrations, and slope of the regression line between log erythropoietin and Hb [11,21], but only one of them showed positive results [11]. It is likely that the increase in erythropoietin mediates improvements in Hb during VA repletion or that VA could form a complex with Fe, keeping it soluble in the intestinal lumen, as well as preventing the inhibitory factors on Fe absorption [30].

Control of VA deficiency and nutritional anemia are amongst the major challenges to all public health and nutrition programs in developing countries, especially where low consumption of VA-rich and Fe-rich food has been reported. VA supplementation has a positive impact on hematological and biochemical indicators of Fe status, and the simultaneous administration of both nutrients (VA and Fe) has been demonstrated as being even more effective in groups with VA deficiency. Nevertheless, micronutrient supplementation/fortification programs in regions where VA and Fe deficiencies are endemic should take into account this interaction.

## 4. Conclusions

Most of the clinical trials included in this review showed an impact of VA supplementation/fortification on anemia, but the simultaneous use of VA and Fe was more effective than the use of any of these micronutrients alone. However, not all the studies assessed the vitamin A status of the population at baseline and included a reasonable number of iron parameters. Additionally, assessment of Hb variants, inflammatory markers and anemia of chronic inflammation would be important in some of the studies.

The precise relationship between VA and Fe depends on many factors. Studies involving different populations are necessary to elucidate the interaction between them, especially on Fe absorption and modulation of erythropoiesis. Considering that multiple micronutrient deficiencies often coexist, and that several micronutrients play an important role in the etiology of nutritional anemia, we also suggest further investigations to assess the effect of combined micronutrient regimens on anemia control.

## Figures and Tables

**Figure S1:**
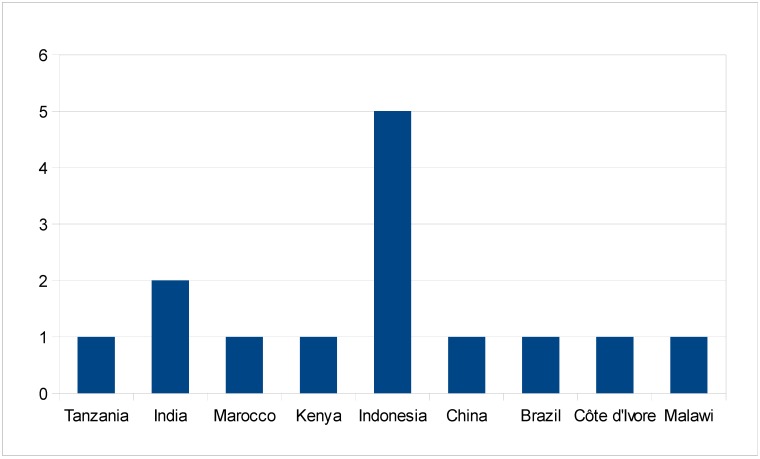
Distribution of the 14 reviewed studies according to country of origin.
